# Cell Death Induction and Protection by Activation of Ubiquitously Expressed Anion/Cation Channels. Part 1: Roles of VSOR/VRAC in Cell Volume Regulation, Release of Double-Edged Signals and Apoptotic/Necrotic Cell Death

**DOI:** 10.3389/fcell.2020.614040

**Published:** 2021-01-12

**Authors:** Yasunobu Okada, Ravshan Z. Sabirov, Kaori Sato-Numata, Tomohiro Numata

**Affiliations:** ^1^National Institute for Physiological Sciences, Okazaki, Japan; ^2^Department of Physiology, School of Medicine, Aichi Medical University, Nagakute, Japan; ^3^Department of Physiology, Kyoto Prefectural University of Medicine, Kyoto, Japan; ^4^Laboratory of Molecular Physiology, Institute of Biophysics and Biochemistry, National University of Uzbekistan, Tashkent, Uzbekistan; ^5^Japan Society for the Promotion of Science, Tokyo, Japan; ^6^Department of Physiology, School of Medicine, Fukuoka University, Fukuoka, Japan

**Keywords:** cell volume regulation, apoptotic cell death, necrotic cell death, VSOR/VRAC, glutamate release, GSH release, cisplatin resistance, programmed necrosis

## Abstract

Cell volume regulation (CVR) is essential for survival and functions of animal cells. Actually, normotonic cell shrinkage and swelling are coupled to apoptotic and necrotic cell death and thus called the apoptotic volume decrease (AVD) and the necrotic volume increase (NVI), respectively. A number of ubiquitously expressed anion and cation channels are involved not only in CVD but also in cell death induction. This series of review articles address the question how cell death is induced or protected with using ubiquitously expressed ion channels such as swelling-activated anion channels, acid-activated anion channels and several types of TRP cation channels including TRPM2 and TRPM7. The Part 1 focuses on the roles of the volume-sensitive outwardly rectifying anion channels (VSOR), also called the volume-regulated anion channel (VRAC), which is activated by cell swelling or reactive oxygen species (ROS) in a manner dependent on intracellular ATP. First we describe phenotypical properties, the molecular identity, and physical pore dimensions of VSOR/VRAC. Second, we highlight the roles of VSOR/VRAC in the release of organic signaling molecules, such as glutamate, glutathione, ATP and cGAMP, that play roles as double-edged swords in cell survival. Third, we discuss how VSOR/VRAC is involved in CVR and cell volume dysregulation as well as in the induction of or protection from apoptosis, necrosis and regulated necrosis under pathophysiological conditions.

## Introduction

For the survival of animal cells, control of their cell volume is essential, since the water permeability of cell membranes is high enough to allow passive water fluxes in response to changes in the extracellular and/or intracellular osmolarity under both physiological and pathological situations (see Books: Okada, 1998; Lang, 2006). Animal cells cope with osmotic cell swelling by the regulatory volume decrease (RVD) and with osmotic cell shrinkage by the regulatory volume increase (RVI) attained by losing KCl and gaining NaCl from intracellular and extracellular solutions, respectively (Lang et al., 1998; Okada, 2004; Hoffmann et al., 2009). Among a large variety of ion channels and transporters, most ubiquitously expressed anion and cation channels ought to predominantly participate in the mechanisms of cell volume regulation (CVR), because this fundamental function is conserved throughout evolution in animal cells irrespective of cell types for cell survival. These ubiquitous volume-regulatory ion channels include swelling-activated anion channels and stretch-activated TRP cation channels as well as cell shrinkage-activated cation channels, that is called the hypertonicity-induced cation channel (HICC) (Wehner et al., 2003b). These volume-regulatory ion channels also play protective roles against cell injury and death caused by osmotic stress. The most ubiquitous swelling-activated, volume-regulatory anion channel is called the volume-sensitive outwardly rectifying anion channel (VSOR) (Okada, 1997), the volume-regulated anion channel (VRAC) (Nilius et al., 1997) or the volume-sensitive organic osmolyte/anion channel (VSOAC) (Strange et al., 1996). Here, we call VSOR/VRAC or simply VSOR.

Dysfunction of CVR leads to cell death. Actually, persistent cell shrinkage and swelling are major hallmarks of apoptotic and necrotic cell death, and called the apoptotic volume decrease (AVD) (Maeno et al., 2000) and the necrotic volume increase (NVI) (Okada et al., 2001), respectively. AVD and NVI are brought about by water fluxes driven by net loss of cellular KCl and net gain of ambient NaCl, respectively. Thus, cell death is initiated by dysfunction or impairments of CVR mechanisms. Common pathological situations are injuries caused by hypoxia or ischemia and that followed by re-oxygenation or reperfusion, and they cause a variety of tissue stress including not only osmotic perturbation but also production of reactive oxygen species (ROS) and acidic overload. ROS are known to activate VSOR, TRPM7 and TRPM2. Extracellular acidification is known to directly activate one type of anion channel which is called the acid-sensitive outwardly rectifying anion channels (ASOR) (Wang et al., 2007) or the proton-activated chloride channel (PAC) (Yang et al., 2019a). Acidity also rapidly augments TRPM7 cation channel activity (Jiang et al., 2005; Numata et al., 2019). Thus, altered activities of VSOR/VRAC and ASOR/PAC anion channels as well as of TRPM2 and TRPM7 cation channels are involved in dysfunction of CVR that eventually leads to cell death. In the present article, we review the roles of VSOR activity (in Part 1), as well as of ASOR/PAC, TRPM2, and TRPM7 activities (in Part 2) in cell death induction and protection.

## Phenotypic Pore Properties and Molecular Identity of VSOR/VRAC

### Phenotypical Properties of VSOR/VRAC Currents

Among a number of types of mammalian anion channels, VSOR and the maxi-anion channel (Maxi-Cl) are activated by cell swelling and thereafter involved in RVD, thus both being called volume-regulatory anion channels (Okada et al., 2018). The functional expression of VSOR was first discovered in 1988 independently by two groups (Cahalan and Lewis, 1988; Hazama and Okada, 1988). Its phenotypical properties were fully clarified by a large number of groups (Strange et al., 1996; Nilius et al., 1997; Okada, 1997), and can be summarized as volume-sensitive, mildly outward-rectifying, non-hydrolytically ATP-dependent anion channels with exhibiting an intermediate single-channel conductance, low electric-field anion selectivity (of Eisenman’s sequence I), sensitivity to intracellular free Mg^2+^, and inactivation kinetics at large positive potentials (Okada et al., 2019b). VSOR was found to be activated not only by cell swelling but also by ROS independently by three groups (Browe and Baumgarten, 2004; Shimizu et al., 2004; Varela et al., 2004) and by a rise of nano-domain intracellular free Ca^2+^ induced by G protein-coupled receptor (GPCR) stimulation (Akita and Okada, 2011; Akita et al., 2011).

### Molecular Identities of VSOR/VRAC Core Components

Since the discovery of VSOR activity in 1988, its molecular entity had not been uncovered for a quarter of a century, despite much efforts of proposing and disproving a number of false-positive candidates including *P*-glycoprotein, pIcln, ClC-3, Best1 and some TMEM16 (ANO) members especially TMEM16F (ANO6), as summarized in recent review articles (Pedersen et al., 2016; Okada et al., 2018, 2019b). At last, through unbiased genome-wide approaches, LRRC8A was recently identified as the core component of human VSOR independently by two groups (Qiu et al., 2014; Voss et al., 2014). This fact was subsequently confirmed to hold for VSOR endogenously expressing in zebrafish (Yamada et al., 2016), mouse (Okada et al., 2017), rat (Elorza-Vidal et al., 2018), and insect (Kern et al., 2019). Furthermore, Jentsch’s group elucidated that functional VSOR activity requires LRRC8A together with LRRC8C, LRRC8D and/or LRRC8E (Voss et al., 2014). Sequential co-immunoprecipitation studies evidenced physical interactions between LRRC8A, LRRC8C and LRRC8E (Lutter et al., 2017). In fact, a recent cryo-electron microscope (cryo-EM) study demonstrated the hexameric structure of LRRC8A together with LRRC8C (Deneka et al., 2018). However, it must be noted that there may be some missing component or subcomponent other than LRRC8 members, in light of the following facts. (1) Double overexpression of LRRC8A and LRRC8C/8D/8E never increased VSOR currents over the endogenous level in HEK293 and HCT116 cells (Voss et al., 2014) and HeLa cells (Okada et al., 2017). (2) Overexpression of LRRC8A *plus* LRRC8D/8E in cisplatin-resistant KCP-4 cells, that are largely deficient in VSOR activity, failed to restore VSOR currents up to the level in its parental cisplatin-sensitive KB cells (Okada et al., 2017). (3) Different cell types with similar LRRC8 expression levels showed differences in VSOR activities (Okada et al., 2017). (4) The activity of channels reconstituted with LRRC8A *plus* LRRC8D/8E was found to be independent of intracellular ATP (Syeda et al., 2016), the fact being at variance with native VSOR activity that is requisitely dependent on intracellular ATP (Jackson et al., 1994; Oiki et al., 1994). (5) Furthermore, the channel reconstituted with purified LRRC8A *plus* LRRC8D/8E was not activated by inflation-induced membrane expansion (Syeda et al., 2016), the fact being contradictory to a known fact that VSOR can be activated by pressure-induced cell inflation (Hagiwara et al., 1992; Doroshenko, 1998; Best and Brown, 2009). In place of LRRC8 members, more recently, Tweety homologs (TTYH1, TTYH2, and TTYH3) were proposed as the VSOR core molecules in mouse astrocytes by Han et al. (2019). Subsequently, TTYH1 and TTYH2 were reported to serve as VSOR, in a manner independent of LRRC8A, in human cancer cells including gastric SNU-601, hepatic HepG2 and colonic LoVo cells by Bae et al. (2019). Our data also showed that hypotonicity-induced VSOR currents were significantly suppressed by siRNA-mediated triple knockdown of TTYH1, TTYH2 and TTYH3 in human cervical HeLa cells (Okada et al., 2020), suggesting an involvement of TTYHs in the regulation or formation of VSOR. However, it must be pointed out that studies with making gene knockout and channel reconstitution of TTYH1, TTYH2, and TTYH3 are still missing to firmly support the essential roles of TTYHs in the VSOR/VRAC channel formation. At moment, we need to know as to whether TTYHs can physically interact with LRRC8s and whether the VSOR activity can be restored by overexpression of TTYHs into cells in which all LRRC8s are knocked out. Also, it must be stressed that it is still not definitely determined whether LRRC8 and/or TTYH form the VSOR pore *per se*, as summarized elsewhere (Okada et al., 2018; Okada, 2019), because drastic alterations in the anion selectivity Eisenman’s sequence and/or in the anion/cation permeability ratio have not as yet been shown to be elicited by any charge-modifying, especially charge-reversing, mutations at their putative pore-forming regions.

### Physical Dimensions of the VSOR/VRAC Pore

The pore size of native VSOR channel was evaluated by three different methods. First, the cut-off size of the organic anions with limited permeability yielded the radius (*R*) of 0.37 nm; when the same data were approximated using the excluded area theory with taking frictional forces into account, the value of 0.58 nm was obtained (Nilius et al., 1999; Nilius and Droogmans, 2003). Second, the cross-sectional radius of the VSOR pore was estimated from the experiments with calixarenes, basket-shaped compounds, acting as permeant blockers, and was found to be 0.57–0.71 nm (Droogmans et al., 1998, 1999). Third, a value of 0.63 nm was obtained by the non-electrolyte partitioning method using non-charged polyethylene glycols (Ternovsky et al., 2004). Thus, based on three different and unrelated methods, it is concluded that the native VSOR pore has a functional radius of 0.6–0.7 nm at the narrowest portion of the ion-conducting pathway.

**FIGURE 1:**
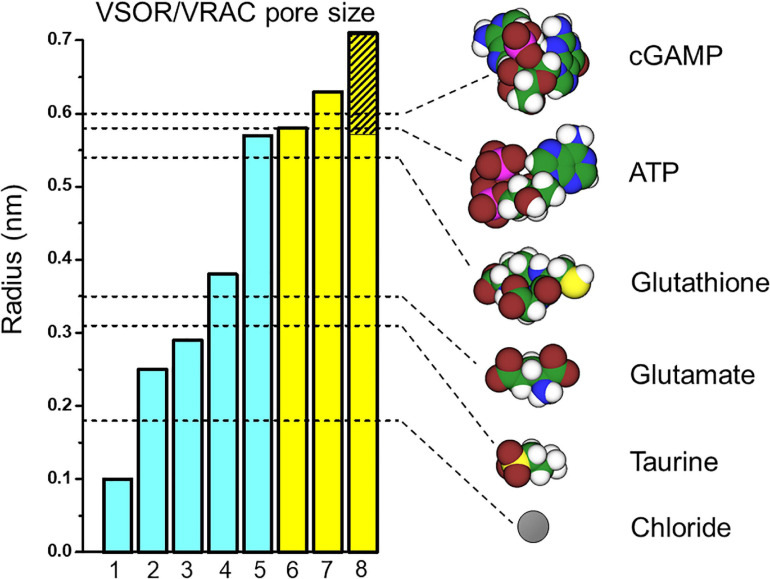
VSOR/VRAC pore size and dimensions of the signaling molecules released via VSOR/VRAC. The pore radii depicted as the bar graphs were taken from: 1 – Kefauver et al., 2018; 2 – Kern et al., 2019; 3 – Deneka et al., 2018; 4 – Kasuya et al., 2018; 5 – Nakamura et al., 2020; 6 – Nilius et al., 1999; Nilius and Droogmans, 2003; 7 – Ternovsky et al., 2004; 8 – Droogmans et al., 1998, 1999. The values for bars #1 and #2 were deduced from the graphs of the pore radius plotted against the distance along the pore axis by the cited authors. Other values are as given by the cited authors. The hatched part of bar #8 indicates lower and upper limits given by the authors. Blue and yellow bars represent the radii structurally evaluated by cryo-EM studies for LRRC8A/D homohexamers and those functionally estimated for the native VSOR channel by electrophysiological studies, respectively. The unhydrated radii of the organic anions (shown as dashed lines) were calculated as a geometric mean of molecular dimensions produced using Molecular Modeling Pro computer software (Norgwyn Montgomery Software Inc., North Wales, PA, United States). Values for ATP and glutamate were reported previously (Sabirov and Okada, 2005). Color coding: C, green; H, white; O, red; N, blue; P, purple; S, yellow; Cl, gray.

Cryo-EM studies of the recombinant LRRC8 paralogs combined with single particle analysis generated a series of spectacular 3D-structures of the VSOR channel (Deneka et al., 2018; Kasuya et al., 2018; Kefauver et al., 2018; Kern et al., 2019; Nakamura et al., 2020), which produced pore dimensions along the central axis of the channel, as depicted together with the above functional radii in Figure 1 (left panel). According to the structure of the mouse LRRC8A homohexamer reported by Deneka et al. (2018), the pore is ∼10 nm long but not uniform: it begins with a wide extracellular vestibule with a radius of ∼0.8 nm followed by a constriction with *R* ∼ 0.29 nm located at about 1.5 nm from the entrance; then the pore widens up to *R* ∼ 1.6 nm around the TM region and ends with an intracellular vestibule with a radius of ∼0.7 nm. The structure of human homohexameric LRRC8A was found to have a similar extracellular vestibule of ∼0.74 nm, but the constriction, the transmembrane (TM) region and the intracellular vestibule were wider with radii of ∼0.38, 2.54, and 1.13 nm, respectively (Kasuya et al., 2018). It is plausible that the narrowest constriction part of the pore serves as the selectivity filter, which restricts the passage of ions and osmolytes. The radius of constriction was smallest (*R* ∼ 0.1 nm) in the structure reported by Kefauver et al. (2018). It should be noted that the ionic strength conditions and lipid environments significantly affect the packaging of the channel protein generating tighter structures with a narrower pore or more relaxed structures with a wider pore (Deneka et al., 2018; Kasuya et al., 2018; Kefauver et al., 2018; Kern et al., 2019). The largest radius of the constriction part (*R* ∼ 0.57 nm), which is close to the functional radius of the native VSOR pore, was reported for the homohexameric channel formed by human LRRC8D (Nakamura et al., 2020). This paralog is known to play an important role in the permeability for charged and non-charged organic osmolytes (Lutter et al., 2017) including Pt-based anti-cancer drugs (Planells-Cases et al., 2015) and antibiotic blasticidin S (Lee et al., 2014). As seen in Figure 1 (left panel), the radii structurally evaluated by cryo-EM observations are smaller than those functionally estimated by electrophysiological recordings. In this regard, it must be pointed out that the cryo-EM structural radii correspond to homohexamer of LRRC8A or LRRC8D but not the native VSOR-forming heterohexamer of LRRC8A+LRRC8C/D/E. Also, one cannot rule out a possibility that the cryo-EM structure may represent the closed state, but not the open state, of the channel.

Taken together, it appears that the native heterohexameric channel composed of various combinations of LRRC8 paralogs in native lipid environments should have an effective radius of 0.6–0.7 nm in the open state. As depicted in Figure 1 (right panel), this size would thus allow passage of not only chloride (*R* ∼ 0.18 nm) but also of taurine (*R* ∼ 0.31 nm), excitatory amino acids such as aspartate (*R* ∼ 0.34 nm) and glutamate (*R* ∼ 0.35 nm) very freely, and also ATP, ADP and UTP (*R* ∼ 0.53–0.61 nm) in a very limited manner (for more osmolytes see Table 2 in Sabirov and Okada, 2005).

## Roles of VSOR/VRAC in Release of Organic Signals for Cell Death Induction/Protection

### Role of VSOR/VRAC in Release of Excitotoxic Glutamate

Glutamate is the principal and most important excitatory neurotransmitter in the vertebrate nervous system under physiological conditions (Meldrum, 2000). Glutamate is released from neurons by the vesicular exocytosis mechanism into the synaptic cleft, and then is cleared by a Na^+^-dependent reuptake mechanism, which keeps the extracellular glutamate at concentrations below the activation threshold for glutamate receptors. The extracellular space normally occupies about one quarter of the total brain volume, but it decreases down to 12–17% during over-excitation by repetitive stimulation or even down to ∼5% upon ischemia by redistribution of water between the extracellular and intracellular space leading to swelling of neurons and astrocytes (Nicholson, 2005; Syková, 2004). Brain edema also occurs as a result of stroke, trauma, brain tumors, systemic viral and bacterial infections (Kimelberg, 2004, 2005; Kimelberg et al., 2004). Swollen neurons and astroglia massively release glutamate, which in turn induces excitotoxicity – neuronal death caused by overexcitation of glutamatergic receptors (Kimelberg et al., 1990; Strange et al., 1996; Liu et al., 2006, 2009; Akita and Okada, 2014; Hyzinski-García et al., 2014; Planells-Cases et al., 2015; Lutter et al., 2017; Schober et al., 2017; Okada et al., 2019a). Thus, glutamate exhibits double-edged functions in the brain.

The putative pathways for glutamate release from swollen cells include: (i) Ca^2+^-dependent vesicular exocytosis, (ii) Na^+^-dependent glutamate transporters functioning in a reverse mode, and (iii) ion channel-mediated conductive release through gap junction hemichannels and/or chloride channels including VSOR and the maxi-anion channel (Phillis and O’Regan, 2003; Evanko et al., 2004; Parpura et al., 2004; Mongin, 2007, 2016; Sabirov et al., 2016; Osei-Owusu et al., 2018; Wilson and Mongin, 2018; Chen et al., 2019; König and Stauber, 2019; Okada et al., 2019a, b; Strange et al., 2019).

First evidence for conductance of VSOR to amino acids such as glutamate was provided by Banderali and Roy (1992). The effective radius of the native VSOR is sufficient to pass glutamate and aspartate (Sabirov and Okada, 2005) (Figure 1). Indeed, the astrocytic channel was permeable to glutamate (Glu) with *P_*Glu*_/P_*Cl*_* = 0.15 (Liu et al., 2006), close to *P_*Glu*_/P_*Cl*_* = 0.14 found in C6 glioma cells (Jackson et al., 1994). These values are within the range of *P_*Glu*_/P_*Cl*_* ∼ 0.06–0.2 reported for many other cells (Banderali and Roy, 1992; Chan et al., 1994; Roy and Banderali, 1994; Roy, 1995; Arreola et al., 1996; Basavappa et al., 1996; Boese et al., 1996; Levitan and Garber, 1998; Schmid et al., 1998; Carpaneto et al., 1999; Sakai et al., 1999; Schlichter et al., 2011). A somewhat higher glutamate permeability was recently reported for cultured primary astrocytes (∼0.3) (Yang et al., 2019b).

Pharmacological studies using VSOR blockers indicated that osmotic swelling or oxygen-glucose deprivation induces massive VSOR-mediated release of glutamate (Liu et al., 2006, 2009; Rudkouskaya et al., 2008; Hyzinski-García et al., 2011; Bowens et al., 2013) and aspartate (Kimelberg et al., 1990; Mongin et al., 1999; Mongin and Kimelberg, 2002; Haskew-Layton et al., 2005, 2008; Mongin and Kimelberg, 2005; Abdullaev et al., 2006; Bowens et al., 2013; Hyzinski-García et al., 2014) from primary cultured astrocytes *in vitro*. Stimulation with hypotonic solution or zymosan was found to induce release of excitatory amino acids from rat microglia in a manner sensitive to VSOR blockers including the most selective blocker DCPIB (Harrigan et al., 2008). An inflammatory initiator bradykinin, which is also released upon brain ischemia, triggers ROS production and thus induces VSOR activation in astrocytes, thereby releasing glutamate therefrom (Haskew-Layton et al., 2005; Liu et al., 2009). Osmotic cell swelling and cell swelling associated with spreading depression also caused massive release of excitatory amino acids from brain slices in a manner sensitive to VSOR blockers (Basarsky et al., 1999; Bothwell et al., 2001). Release of excitatory amino acids, which is inhibited by VSOR blockers, was observed *in vivo* in animal models of global and focal ischemia (Phillis et al., 1997, 1998; Seki et al., 1999; Kimelberg et al., 2000, 2003; Feustel et al., 2004; Zhou J.J. et al., 2020). VSOR blockers protected neurons from delayed neuronal death after transient forebrain ischemia (Abdullaev et al., 2006; Inoue et al., 2007).

After a hetero-multimer of LRRC8 family proteins was identified as the core component of VSOR, a causative relationship between VSOR and swelling-induced release of excitatory amino acids was verified by siRNA-mediated LRRC8A knockdown (Hyzinski-García et al., 2014; Sørensen et al., 2014) and by LRRC8A knockout (Lutter et al., 2017; Yang et al., 2019b) and also by LRRC8D knockout (Lutter et al., 2017) and its knockdown (Schober et al., 2017).

It should be noted that VSOR is not the only anion channel contributing to the glutamate release. Involvements of at least two different pathways in osmolyte release were suggested based on their time courses, Ca^2+^ dependence, and pharmacological profiles (Mongin et al., 1999; Franco et al., 2001; Mongin and Kimelberg, 2002; Pasantes-Morales et al., 2002; Netti et al., 2018). Astrocytes express high levels of the glutamate-permeable (*P_*Glu*_/P_*Cl*_* = 0.21) maxi-anion channel which accounts for about half of the hypotonicity-induced and one third of the ischemia-induced glutamate release (Liu et al., 2006). Note that this channel does not contribute to the bradykinin-induced glutamate release, which is solely mediated by VSOR activated by ROS and a Ca^2+^ nanodomain-related mechanism in astrocytes (Liu et al., 2009; Akita and Okada, 2011, 2014).

### Role of VSOR/VRAC in Release of Natural Antioxidant Glutathione

The tripeptide glutathione (γ-L-glutamyl-L-cysteinylglycine: GSH) is the most prevalent and ubiquitous constituent of cytosol and is involved in many cellular processes such as antioxidant defense, drug detoxificaion, cell signaling, cell metabolism and proliferation (Meister, 1995; Hammond et al., 2001; Wu et al., 2004). Over 98% of GSH inside the cells exists in its reduced monomeric form at the concentrations of 1–10 mM depending on cell types (Meister and Anderson, 1983; Meister, 1995). GSH is synthesized inside the cells and degraded exclusively outside in the process termed γ-glutamyl cycle. Extracellularly, GSH exists at micromolar levels and protects tissues and cells from oxidative stress: the lung epithelium upon intensive breathing, heart and brain cells during ischemia-reperfusion. Transmembrane delivery of GSH is an important step in the γ-glutamyl cycle; it is performed through the activity of transporters, such as ABCC/MRP (Minich et al., 2006; Ballatori et al., 2009), SLCO/OATP family (Briz et al., 2006; Franco and Cidlowski, 2006), and SLC22A/OAT group transporters (Garcia et al., 2011). Since the GSH molecule bears one net negative charge, its release is caused by activation of conductive pathways, such as CFTR (Linsdell and Hanrahan, 1998; l’Hoste et al., 2010) with a functional pore radius of ∼0.7 nm (Krasilnikov et al., 2011) and gap junction hemichannel (Tong et al., 2015). GSH efflux is known to be a prerequisite to apoptosis induction (Ghibelli et al., 1998; Franco and Cidlowski, 2006; Circu et al., 2009). Thus, GSH release exhibits double-edged actions by playing an anti-oxidant cell-protective role on the extracellular side but a cell death-inducing role on the intracellular side.

The radius of GSH molecule (0.52-0.56 nm) (Sabirov et al., 2013) is slightly less than the effective VSOR pore size (Figure 1, right panel), and, therefore, it is expected that this channel could serve as a pathway for GSH release. Immature thymic lymphocytes exhibit robust RVD ability upon hypotonic stimulation and express high levels of VSOR activity (Kurbannazarova et al., 2003, 2011). As expected, VSOR in rat thymocytes was permeable to this anionic tripeptide with the permeability ratio *P_*G*__*SH*_/P_*Cl*_* of ∼0.1 for outward (efflux) and 0.32 for inward (influx) directions, and this permeability was sufficient to provide the observed GSH release rate of ∼6 attomol/cell/min, which occurred predominantly by a diffusion mechanism and in a DCPIB-sensitive manner (Sabirov et al., 2013). Kidney epithelial cells, HEK293 and HK-2, were shown to express GSH-conductive VSOR with *P_*G*__*SH*_/P_*Cl*_* ∼0.1 (efflux) and exhibited massive swelling-induced GSH release, which was inhibited by DCPIB and not observed LRRC8A-knockout HEK293 cells (Friard et al., 2019). The VSOR activation and associated GSH release were observed in HK-2 cells under isotonic conditions upon exposure to the pleiotropic growth factor TGFβ1 and was essential for the epithelial-to-mesenchymal transition (Friard et al., 2019).

### Role of VSOR/VRAC in ATP Release

Release of adenosine triphosphate (ATP) is a key event in powerful purinergic signaling in most animal tissues (Burnstock, 2012). Released ATP facilitates RVD via stimulation of P2Y receptors (Dezaki et al., 2000; Braunstein et al., 2001; Varela et al., 2010; Islam et al., 2012; Espelt et al., 2013). ATP and glutamate are two major gliotransmitters, which not only modulate synaptic transmission but also protect and repair neuronal tissues after damage. In the heart, extracellular ATP is protective in ischemia-reperfusion damage (Ninomiya et al., 2002; Wee et al., 2007; Burnstock and Pelleg, 2015). It is also known to be degraded by ecto-ATPases to adenosine, which is, in turn, a well-established cardio-protector (Burnstock and Pelleg, 2015). Released ATP produces not only beneficial effects but also detrimental effects depending on cell types and situations. For example, ATP is known to act as a danger signal in a variety of neurological diseases (Franke et al., 2012), brain trauma, hypoxia/ischemia and epilepsy-associated seizures (Rodrigues et al., 2015), and inflammation responses activating apoptosis and autophagy in oxidative conditions (Linden et al., 2019).

ATP is an anion bearing 2–4 negative charges depending on binding to Mg^2+^ and protonation (see Table 1 in Sabirov and Okada, 2005). Its calculated effective radius (0.56–0.61 nm: see Table 2 in Sabirov and Okada, 2005) is compatible with the possible conductive transport via VSOR (Figure 1). Consistent with this possibility, a voltage-dependent open-channel blockage of VSOR by ATP added from the extracellular side (Jackson and Strange, 1995; Tsumura et al., 1996) was found to be relieved at large positive voltages due to translocation of the blocker to the opposite side of the membrane by Hisadome et al. (2002) in accord with the permeating blocker mechanism (Droogmans et al., 1998, 1999). VSOR inhibitors glibenclamide, verapamil, tamoxifen, and fluoxetine suppressed the hypotonicity-induced ATP release in these cells, suggesting that this pathway is used by the nucleotide to exit the cells. More recently, hypotonicity-induced ATP release from HEK293 cells and primary cerebellar granule neurons was observed to be sensitive to treatment with DCPIB and shRNA for LRRC8A, suggesting an involvement of VSOR in the ATP release pathway (Dunn et al., 2020).

Cultured neonatal cardiac myocytes (Dutta et al., 2004) and myocytes isolated from mature left ventricles (Dutta et al., 2008) responded with ATP release to osmotic stress and chemical ischemia. However, this process had a pharmacological profile inconsistent with the role of VSOR but closer to the profile of the maxi-anion channel (Sabirov et al., 2016; Okada et al., 2019a, b). A similar conclusion was made for the ATP release from primary cultured astrocytes (Liu et al., 2008a, b) and from mammary C127 cells (Sabirov et al., 2001). There remains a possibility, thus, that contribution of the channel to the net ATP release is different in different cell types depending on the paralog combinations, since conductive properties of VSOR depends on subunit composition of the LRRC8 hexamers (Syeda et al., 2016). Also, it is feasible that LRRC8 may somehow be involved in regulation of other ATP-releasing pathways, because LRRC8A is known to interact with other plasmalemmal proteins (Benedetto et al., 2016; Choi et al., 2016; Fujii et al., 2018) often via their LRR motifs (Kobe and Kajava, 2001).

### Roles of VSOR/VRAC in Release of Other Signaling Molecules

Taurine (2-aminoethanesulfonic acid) is one of the most abundant intracellular osmolytes. The cytosolic concentration of taurine in the brain neurons and astrocytes is usually 10-20 mM (Walz and Allen, 1987) and may reach as high as 50 mM (Voaden et al., 1977; Huxtable, 1982), and its release has long been considered as one of the key molecular events in volume regulation (Oja and Saransaari, 2017). Taurine with its radius of ∼0.31 nm is well-suited for transport via VSOR (Figure 1), and the process of osmosensitive taurine release is one of the well-documented functions of the LRRC8/VSOR (Jentsch et al., 2016; König and Stauber, 2019; Okada et al., 2019b). Both LRRC8A and LRRC8D are known to be required for hypotonicity-induced taurine release (Planells-Cases et al., 2015; Lutter et al., 2017). Once released, taurine acts on glycine and GABA receptors as a co-agonist, resulting in reduction of neuronal firing and protection from over-excitation (Ye et al., 2013; Oja and Saransaari, 2017).

GABA (gamma-aminobutyric acid) is also released from HEK293 cells via VSOR in an LRRC8D- and LRRC8E-dependent manner (Lutter et al., 2017). LRRC8A deletion and silencing abolished GABA release from mouse and human pancreatic β cells (Menegaz et al., 2019). Since GABA has strong protective and regenerative effects on β cells (Fiorina, 2013), it is likely that an impairment of VSOR-mediated GABA release may participate in etiology of diabetes mellitus. Actually, Type-1 and -2 diabetic islets of human patients were shown to exhibit disrupted secretion of GABA (Menegaz et al., 2019).

Cyclic guanosine monophosphate-adenosine monophosphate (2’3’cGMP-AMP or cGAMP) is synthesized by an enzyme cyclic cGAMP synthase, which senses double-stranded DNA in infected and malignant cells; it is then transferred to the neighboring cells either by gap junctions or by a release-uptake mechanism to trigger interferon production as the cell/host defense against DNA viral infection and other malignancies. It was recently demonstrated that the cGAMP release occurs via LRRC8A/LRRC8E-containing VSOR (Zhou C. et al., 2020). The calculated size of cGAMP (*R* ∼ 0.6 nm) is compatible with the size of VSOR pore (Figure 1). Thus, it is likely that VSOR is involved in the cell/host defense against DNA virus by releasing cGAMP.

## Roles of VSOR/VRAC in Cell Volume Regulation and Cell Death Induction/Protection

### Cell Volume Regulation in Mammalian Cells

**FIGURE 2:**
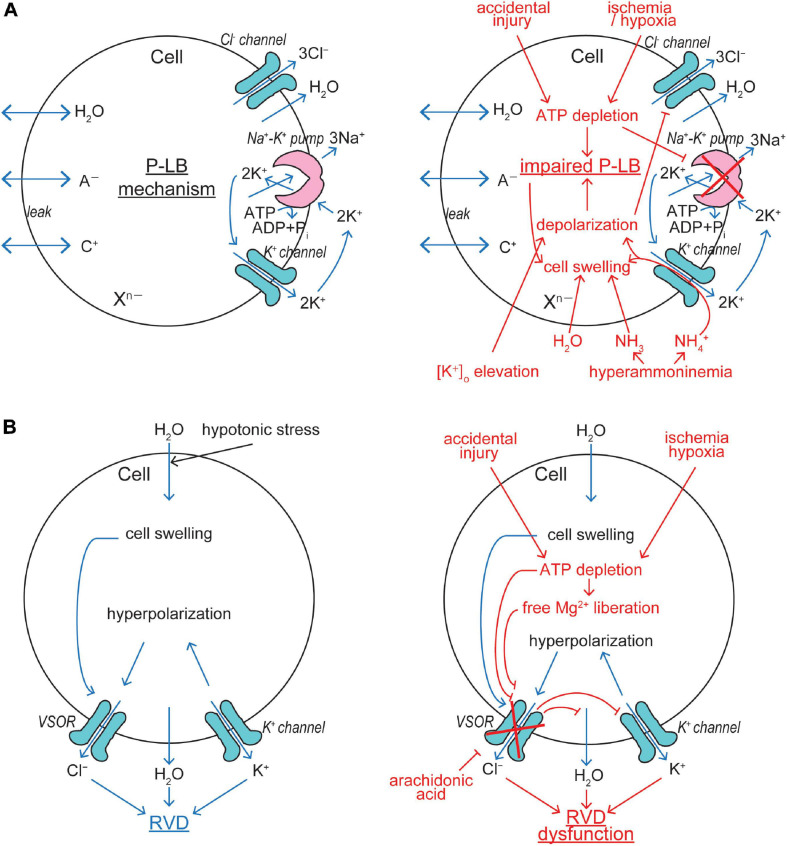
VSOR/VRAC involvements in cell volume regulation/dysregulation. **(A)** The pump-leak balance (P-LB) mechanism coping with steady-state oncotic cell swelling (left panel) and its dysfunction leading to NVI (right panel). The Cl^–^ channel involved in this mechanism is not identified as yet. **(B)** The RVD mechanism coping with non-steady state osmotic cell swelling (left panel) and its dysfunction leading to necrosis (right panel). VSOR plays a key role in this mechanism by sensing cell swelling (See the text for details).

Cell volume regulation is physiologically essential for the cell survival with exhibiting normal functions, and an optimal cell size is likely to be prerequisite to a particular cell’s function. CVR dysfunction is also pathophysiologically important, because sizable changes in the plasma osmolarity are known to often be coupled to a variety of diseases and iatrogenic outcomes (see Table 1 in Okada et al., 2019a). Even under physiological normotonic conditions, the volume of cells is subjected to alterations because of steady-state physicochemical osmotic load and of non-steady state physiological cell activity-dependent fluctuations in intracellular osmolarity (Okada, 2004). The former physicochemical load is caused by the intracellular presence of large numbers of polyvalently anionic macromolecules (X^*n*–^) which are membrane-impermeable (Figure 2A). Such fixed negative charges attract membrane-permeable inorganic cations (C^+^) to the cytosol, and then the cation entry should drive the entry of membrane-permeable inorganic anions (A^–^) due to the electroneutral restraint. However, the inorganic anion entry should be resisted by fixed macromolecular anions by electrostatic repulsion. The resultant equilibrium, called the Gibbs–Donnan equilibrium, brings about increased intracellular osmolarity under normotonic extracellular conditions. This situation can be mathematically expressed as follows, on the basis of Gibbs–Donnan equation: *(RT/F) ln ([C^+^]_*i*_/[C^+^]_*o*_) = (RT/F) ln ([A^–^]_*o*_/[A^–^]_*i*_)* where *i* and *o* stand for the intracellular and extracellular side, respectively. This equation can be transformed to *[C^+^]_*i*_* × *[A^–^]_*i*_* = *[A^–^]_*o*_*^2^ because of *[A^–^]_*o*_* = *[C^+^]_*o*_*. Thus, *[C^+^]_*i*_* + *[A^–^]_*i*_ > [C^+^]_*o*_ + [A^–^]_*o*_*, because *a* + *b* > 2*c* when *a* × *b* = *c*^2^ in general. Coping with such oncotic osmotic pressure, steady-state volume regulation is attained by the pump-leak balance (P-LB) mechanism (Tosteson and Hoffman, 1960), in which net Na^+^ extrusion is persistently produced by active operation of Na^+^-K^+^ pump with simultaneous operation of K^+^ channels for recycling of K^+^. Electrogenic operation of Na^+^-K^+^ pump and electrogenic K^+^ channel opening produce a negative membrane potential, thereby driving the passive extrusion of intracellular Cl^–^ through some anion channels (Figure 2A, left panel). The resultant reduction of intracellular Cl^–^ concentration compensates for the existence of polyvalently anionic macromolecules. In addition to the steady-state oncotic load, cell activities themselves produce non-steady state osmotic load as above stated. Cell volume changes are thus induced by fluctuations of the cellular osmolarity caused by fundamental physiological cell activities such as transport and metabolism of biological substances (see Table 1 in Okada, 2004). Thus, cells need to quickly readjust their volume, in a non-stationary manner, through volume-regulatory transports of osmolyte and water. Under such anisotonic conditions, animal cells cope with osmotic cell swelling and shrinkage by RVD and RVI mechanisms, respectively. The RVD and RVI events are attained by water efflux and influx driven by the exit of KCl and entry of NaCl, respectively. A variety of volume-regulatory KCl and NaCl transport pathways including ion channels and transporters for both symport and antiport have been listed to be involved in CVR mechanisms in the early 1980s (Grinstein et al., 1984; Hoffmann et al., 1984; Sarkadi et al., 1984a; Lauf, 1985).

### Role of VSOR/VRAC in RVD

Conductive K^+^ and Cl^–^ pathways have been suggested to play predominant roles in RVD mechanisms in animal cells by measuring cell volume changes and ionic fluxes in the 1980s (Grinstein et al., 1982, 1983; Sarkadi et al., 1984b, 1985; Hoffmann et al., 1986; Abdullaev et al., 2006). Direct electrophysiological evidence for parallel activation of K^+^ and Cl^–^ channels was first provided in 1988 in human epithelial cells by applying two-microelectrode voltage/current-clamp tecniques (Hazama and Okada, 1988). A large variety of K^+^ channels preinstalled in the plasma membrane are known to serve as volume-regulatory K^+^ channels in most mammalian cells (Wehner et al., 2003a; Hoffmann et al., 2009). The volume-regulatory Cl^–^ channel activated by osmotic cell swelling was thereafter well characterized by applying patch-clamp techniques and is called VSOR or VRAC (Nilius et al., 1997; Okada, 1997). The fact that VSOR is prerequisitely involved in RVD was shown by observations of RVD inhibition by VSOR blockers in a wide variety of cell types (Hazama and Okada, 1988; Kubo and Okada, 1992; Chan et al., 1994; Fatherazi et al., 1994; Robson and Hunter, 1994; Gschwentner et al., 1995a, b; Nilius et al., 1995; Best et al., 1996; Gosling et al., 1996; Shen et al., 1996; Zhang and Jacob, 1996; Leaney et al., 1997; Pasantes-Morales et al., 1997; Bond et al., 1998; Patel et al., 1998; Walker et al., 1999; Mitchell et al., 2002; Al-Nakkash et al., 2004; Parkerson and Sontheimer, 2004; Inoue et al., 2005; Ducharme et al., 2007; Okumura et al., 2009; Chen et al., 2010; Inoue et al., 2010; Cao et al., 2011; Sato et al., 2011; Ponce et al., 2012; Hernández-Benítez et al., 2014; Friard et al., 2017; Trothe et al., 2018). Molecular evidence for the involvement of VSOR in RVD was recently provided by observing inhibition of RVD by LRRC8A knockdown in human HeLa cells (Qiu et al., 2014) and rat astrocytes (Formaggio et al., 2019) as well as by LRRC8A knockout in human HEK293 cells (Voss et al., 2014) and keratinocytes (Trothe et al., 2018). Thus, it is now established that VSOR activity is essentially involved in RVD by cooperating with the activity of K^+^ channels (Figure 2B, left panel).

### Roles of VSOR/VRAC in Apoptosis Induction and Protection

Apoptosis is a physiological type of cell death, by which unnecessary or damaged cells are eliminated, and is classified into two types. One is the intrinsic apoptosis, which is mediated by mitochondria and induced by growth factor withdrawal, oxidative stress, ER stress or DNA damage, and another is the extrinsic apoptosis, which is mediated by stimulation of death receptors such as Fas, TNFR1 and TRAILR1∼4 (Sauler et al., 2019). Normotonic cell shrinkage is a major hallmark of apoptotic cell death (Wyllie et al., 1980) and was termed AVD (Maeno et al., 2000). The AVD induction preceded cytochrome *c* release, caspase-3 activation, DNA laddering and cell death, and all these apoptotic events were prevented by blocking K^+^ and Cl^–^ channels (Maeno et al., 2000). These findings were also reproduced in the process of Fas-induced apoptosis not only in HeLa cells but also lymphoblastoid SKW6.4 cells (Maeno et al., 2012). Since SKW6.4 cells undergo Fas-induced apoptosis without involving mitochondria (Eguchi et al., 1999), we concluded that the AVD induction is an early event independent of the mitochondrial apoptotic signaling pathway (Maeno et al., 2012). In addition, the AVD induction in HeLa cells treated with an intrinsic apoptosis inducer, staurosporine (STS), was found to precede activation of caspase-8 and caspase-9, and overexpression of Bcl-2 failed to inhibit the STS-induced AVD in mouse B lymphoma WEHI-231 cells (Maeno et al., 2012). Also, the AVD induction occurred earlier than apoptotic activation of MAP kinase (Hasegawa et al., 2012). These facts indicate that the AVD induction is an early event independent of activation of initiator caspases and MAP kinases. However, the AVD event further proceeds after activation of caspase-3, as clearly demonstrated by parallel observations of the time courses of AVD and caspase-3 activation (Maeno et al., 2012) (also see Figure 4 in Okada et al., 2019a). Thus, the AVD process is divided by the early phase cell shrinkage independent of caspase activation but dependent on K^+^ and Cl^–^ channels and the late-phase shrinkage dependent on caspase activation. We also demonstrated that apoptotic cells failed to exhibit RVI, and therefore persistence of apoptotic cell shrinkage requires not only AVD induction but also RVI dysfunction (Maeno et al., 2006b). Furthermore, it must be noted that persistent osmotic cell shrinkage experimentally induced *per se* causes induction of apoptotic cell death (Maeno et al., 2006a; Nukui et al., 2006). Taken together, it appears that AVD is the early prerequisite event, which triggers the following executive biochemical processes, for apoptotic cell death.

**FIGURE 3:**
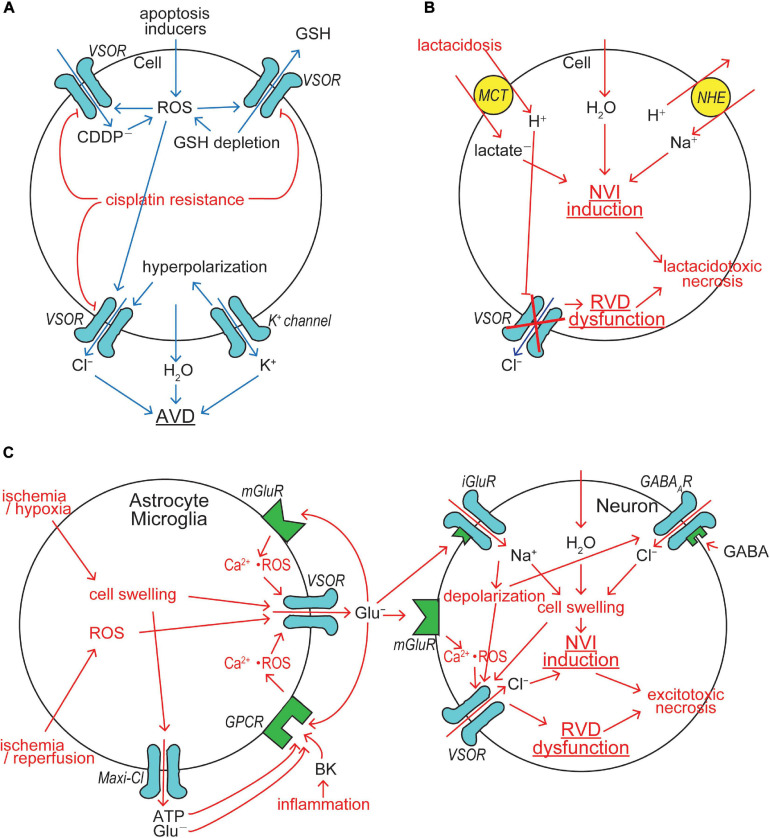
VSOR/VRAC involvements in cell death induction/protection. **(A)** The mechanisms for AVD induction and cisplatin resistance acquisition. VSOR contributes to AVD induction by mediating Cl^–^ efflux, GSH release and cisplatin (CDDP^–^) uptake, whereas VSOR downregulation contributes to cisplatin resistance acquisition. **(B)** The mechanism for lactacidosis-induced NVI in neuronal and glial cells in which VSOR activity is abolished. **(C)** The mechanism for excitotoxicity-induced NVI in neuronal cells in which VSOR rather mediates Cl^–^ influx under depolarization induced by activation of ionotropic glutamate receptor (iGluR) by glutamate released via VSOR from nearby glial cells (See the text for details).

Multiple types of K^+^ channels serve as the AVD-inducing K^+^ release pathway depending on cell types (Burg et al., 2006). On the other hand, we identified, for the first time, that VSOR is responsible for the AVD-inducing Cl^–^ channel in HeLa cells under apoptotic stimulation with STS, Fas ligand (FasL), TNFα+CHX, and ROS directly by patch-clamping (Shimizu et al., 2004). It is noted that increased expression of LRRC8A was recently found to be coupled to FasL-induced apoptosis in smooth muscle cells (Kenagy et al., 2011). Also, caspase activation induced by exposure to STS was found to be strongly reduced in HCT116 cells in which LRRC8A or all LRRC8 genes were disrupted (Planells-Cases et al., 2015). Opening of VSOR channels leads to Cl^–^ efflux driven by hyperpolarization induced by K^+^ channel activation (Figure 3A). Furthermore, we showed the involvement of VSOR in induction of AVD and apoptosis in cardiac myocytes stimulated with STS (Takahashi et al., 2005; Tanabe et al., 2005; Okada et al., 2006) and ROS (Wang et al., 2005). Also, evidence for the involvement of VSOR in ischemia-reperfusion-induced apoptosis was provided in cardiac myocytes (Wang et al., 2005) and for that in delayed neuronal death (DND) of CA1 pyramidal neurons in the hippocampus *in vivo* (Inoue et al., 2007), which is largely due to apoptosis with exhibiting AVD (Okada et al., 2019a) and occurs several days after starting reperfusion following transient forebrain ischemia. Neuronal LRRC8A was judged to contribute to ischemia-induced brain injury, because middle cerebral artery occlusion induced upregulation of LRRC8A expression and augmentation of VSOR currents in hippocampal CA1 neurons derived from control mice but never in those from LRRC8A-knockout mice (Zhou J.J. et al., 2020). Actually, VSOR was found to be involved in induction of AVD and apoptosis in many other cell types by other laboratories (Souktani et al., 2000; Porcelli et al., 2003; d’Anglemont de Tassigny et al., 2004; Liu et al., 2008a, 2013; He et al., 2010; l’Hoste et al., 2010; Shen et al., 2014a, b; Shimizu et al., 2015; Yang et al., 2015; Kumagai et al., 2016; Wang et al., 2017). Thus, it is concluded that VSOR represents the AVD-inducing Cl^–^ channel independent of cell types and apoptotic stimuli.

Cellular GSH depletion or decreased GSH/GSSH (glutathione-disulfide) ratio is known to be a common early event in apoptotic cell death induced by death receptor activation, mitochondrial apoptotic signaling and oxidative stress (Circu and Aw, 2008). For example, GSH depletion sensitizes to TNFα-induced apoptosis in hepatocytes (Matsumaru et al., 2003). GSH depletion and post-translational modifications of proteins through glutathionylation (protein-SSG formation) are critical regulators of apoptosis (Franco and Cidlowski, 2009). Actually, GSH efflux leading to its depletion was shown to be a prerequisite to apoptosis induction (Ghibelli et al., 1998; Franco and Cidlowski, 2006; Circu et al., 2009). The candidates for GSH efflux pathways so far reported were the multidrug resistant protein (MRP), the organic anion transporting polypeptide (OATP), and CFTR (Circu and Aw, 2012). Our study, as noted above, demonstrated that VSOR can serve as a pathway for GSH release (Sabirov et al., 2013). Thus, VSOR activity is likely to be doubly involved in apoptosis induction first by inducing AVD and second by releasing antioxidant GSH (Figure 3A).

The platinum-based drug cisplatin (CDDP) is a widely used anti-cancer drug, which induces apoptotic death in cancer cells after invading the cells. We demonstrated that VSOR activity is involved in induction of AVD and apoptosis in human cancer KB cells stimulated with cisplatin (Ise et al., 2005). In agreement with this observation, LRRC8A expression was found to be increased in human cancer A549 cells exposed to cisplatin (Thorsteinsdottir et al., 2016). Overexpression of LRRC8A was also observed to augment apoptosis induced by another anti-cancer drug, temozolomide, in glioma cells (Yang et al., 2019). Furthermore, cisplatin-induced apoptosis in human HCT116 cells was prevented by gene knockout of LRRC8A, LRRC8D and all members of LRRC8 (Planells-Cases et al., 2015). CDDP is not much lipid-soluble but largely water-soluble (see Figure 5 in Okada et al., 2019a), and therefore its entry needs to be mediated by some channels or transporters. Recently, the cisplatin entry pathway was demonstrated to be provided by the VSOR channel composed of both LRRC8A and LRRC8D (Planells-Cases et al., 2015). In fact, the VSOR pore size was found to be large enough to be permeated by CDDP (see Figure 5 in Okada et al., 2019a).

The acquisition of resistance to cisplatin by cancer cells is the major limitation for cancer chemotherapy with cisplatin. Our study (Lee et al., 2007) demonstrated, for the first time, that protection from cisplatin-induced apoptosis, called cisplatin resistance, is coupled to downregulation of VSOR channel activity in a cisplatin-resistant cancer cell line, KCP-4, which is derived from cisplatin-sensitive parental KB cells (Fujii et al., 1994), suggesting VSOR is an essential factor in cisplatin sensitivity and resistance of the cancer cells. This inference was proven by the observations that cisplatin sensitivity of KCP-4 cells was restored when the VSOR activity was restored by treatment of two different histone deacetylase (HDAC) inhibitors (Lee et al., 2007; Shimizu et al., 2008). An essential role of VSOR in anti-cancer drug resistance was thereafter shown in numbers of other cell types (Poulsen et al., 2010; Min et al., 2011; Yang et al., 2015). This conclusion was molecularly confirmed by recent observations that cisplatin resistance is correlated with reduced expression of LRRC8A in human cancer cells (Planells-Cases et al., 2015; Sørensen et al., 2016a, b; Thorsteinsdottir et al., 2016). Also, temozolomide resistance was found to be associated with downregulation of LRRC8A in glioma cells (Yang et al., 2019). In contrast, cisplatin resistance in KCP-4 cells was recently found to be coupled to VSOR dysfunction due to disruption of actin filaments but not due to decreased expression of LRRC8A (Shimizu et al., 2020). Taken together, it is clear that VSOR is involved in AVD induction and CDDP uptake and that downregulation of VSOR activity causes cisplatin resistance in cancer cells by reducing both AVD-inducing and cisplatin-permeating activities of VSOR (Figure 3A).

### Roles of VSOR/VRAC in Necrosis Induction and Protection

Necrosis is induced by a variety of insults and starts with normotonic cell swelling, called NVI (Okada et al., 2001). Persistence of necrotic cell swelling requires not only NVI induction but also RVD dysfunction (Okada et al., 2004). Necrosis is classified into accidental necrosis, which is induced by injury, ischemia/hypoxia, DNA strand break, hyperammonemia, acidosis, lactacidosis and excitotoxicity, and programmed or regulated necrosis, including necroptosis, pyroptosis and ferroptosis, which is induced by lipopolysaccharide, viral infection, Toll-like receptor activation, and TNF receptor activation in the absence of caspase-8 activity.

Intracellular ATP depletion by ≥80% is a characteristic of necrosis (Simard et al., 2012). This is in contrast to the fact that apoptosis requires ATP generation and is associated with a rise of the intracellular ATP level (Zamaraeva et al., 2005, 2007). Thus, depletion of cellular ATP even switches the form of cell death from apoptosis to necrosis (Eguchi et al., 1997; Leist et al., 1997). Ischemia/hypoxia insults and accidental injury cause NVI induction due to ATP depletion, because resultant inhibition of Na^+^-K^+^ pump impairs the P-LB mechanism which contributes to steady-state CVR (Figure 2A, right panel). In addition, ATP depletion directly and indirectly, via a rise of intracellular free Mg^2+^ liberated from Mg-ATP, inhibits VSOR activity (Oiki et al., 1994; Okada et al., 2019b), thereby causing RVD dysfunction (Figure 2B, right panel). Arachidonic acid, which is produced in the ischemic/hypoxic tissues, was shown to be a very potent blocker for VSOR and thereby abolish RVD (Kubo and Okada, 1992). In the ischemic/hypoxic brain, the extracellular K^+^ concentration is known to largely elevate (Hansen, 1985). Also, extracellular K^+^ accumulation is induced by excessive neuronal activity during epileptic episodes (Heinemann et al., 1986; Walz, 2000; Carmignoto and Haydon, 2012). Elevation of the extracellular K^+^ level results in membrane depolarization which drives Cl^–^ inflow, but not outflow, thereby disrupting the P-LB mechanism and then leading to NVI (Figure 2A, right panel). Hyperammonemia, which is caused by liver diseases, also brings about NVI by impairing the P-LB mechanism, as summarized by Wilson and Mongin (2018), because the K^+^ channel-mediated NH_4_^+^ conductance impairs the K^+^ channel role in this steady-state CVR mechanism not only by provoking depolarization and thus reducing Cl^–^ efflux but also by shunting K^+^ recycling and thus retarding the Na^+^-K^+^ pump operation (Figure 2A, right panel).

Cerebral ischemia/hypoxia, trauma, seizure and spreading depression often result in not only acidosis due to proton liberation from hydrolysis of ATP greater than its synthesis but also lactate accumulation due to enhanced anaerobic glycolysis-fermentation reactions (Siesjö, 1988; Marmarou, 1992). Acidosis coupled to lactate accumulation, that is lactacidosis, causes glial and neuronal cell swelling, cytotoxic brain edema and necrotic death of these brain cells (Kraig et al., 1987; Siesjö, 1988; Staub et al., 1990, 1993). Anionic lactate is taken up with H^+^ by the monocarboxylate transporter (MCT), thereby causing intracellular accumulation of lactate and proton which stimulates the Na^+^/H^+^ antiporter (NHE) and leads to Na^+^ accumulation, thereby causing NVI induction. Accumulated protons, on the other hand, inhibit VSOR activity (Gérard et al., 1998; Sabirov et al., 2000; Kittl et al., 2019), thereby resulting in RVD dysfunction. Lactacidosis-induced NVI induction and RVD dysfunction were actually observed in both neuronal cells (Mori et al., 2002) and glial cells (Nabekura et al., 2003). Persistent cell swelling caused by the NVI induction and RVD dysfunction should finally lead to necrotic cell death, here called lactacidotoxic necrosis, in neuronal and glial cells (Okada et al., 2019a), as depicted in Figure 3B.

Cerebral ischemia, ischemia-reperfusion, stroke, brain trauma, brain inflammation and a number of neurodegenerative disorders or neurogenic diseases often cause massive release of glutamate (Glu^–^) from astrocytes (Olney et al., 1971), and neuronal and glial cell swelling and death, coined excitotoxicity (Olney, 1969), are produced due to exposure to excessive glutamate (Hasbani et al., 1998). Glial glutamate release is mediated by VSOR, as depicted in Figure 3C (left panel). Hypoxia/ischemia-induced swelling in astrocytes releases glutamate largely via VSOR (Abdullaev et al., 2006; Liu et al., 2006; Zhang et al., 2008; Bowens et al., 2013) and also glutamate and ATP via Maxi-Cl channels (Liu et al., 2006). Anoxia-induced glutamate release was inhibited by gene silencing of LRRC8A in astrocytes (Wilson et al., 2019). Astroglial LRRC8A is also required for stroke-induced brain damage, because conditional knockout of LRRC8A protected from ischemic stroke (Yang et al., 2019b). Ischemia causes release of bradykinin (BK), an initial mediator of brain inflammation (Gröger et al., 2005). BK was shown to activate VSOR via stimulation of BK type 2 receptor (BK2) in mouse astrocytes, thereby releasing glutamate (Liu et al., 2009). Since astrocytic VSOR is activated by ATP (Akita et al., 2011) and glutamate (Akita and Okada, 2014) through stimulation of their GPCRs via a signal here called Ca^2+^⋅ROS which represents ROS production mediated by Ca^2+^ nanodomains, glutamate release may also be induced from astrocytes exposed to extracellular ATP and glutamate. Glial glutamate release was also shown to be induced by ROS by activating VSOR in rat astrocytes (Haskew-Layton et al., 2005) and microglia (Harrigan et al., 2008). These activation mechanisms of glutamate-releasing VSOR channels are also summarized in Figure 3C (left panel).

Chronic excitotoxicity may play a role in pathogenesis of a variety of neurodegenerative diseases including amyotrophic lateral sclerosis, Altzheimer’s disease, and Huntington’s disease (Lewerenz and Maher, 2015). Excessive glutamate release from glial cells causes neuronal cell swelling (NVI) and necrotic death by the excitotoxic mechanism. Such acute excitotoxicity is known to be dependent on the entry of Na^+^ and Cl^–^ rather than Ca^2+^ entry into neurons (Rothman, 1985; Olney et al., 1986; Chen et al., 1998; Inglefield and Schwartz-Bloom, 1998). Glutamate-induced stimulation of ionotropic glutamate receptor (iGluR) cation channels causes Na^+^ influx and depolarization in neuronal cells. Ischemia induces release of gamma-aminobutyric acid (GABA) from neurons and astrocytes (Inglefield and Schwartz-Bloom, 1998). Glutamate also induces GABA release from GABAergic neurons (Weiss, 1988; Harris and Miller, 1989; Pin and Bockaert, 1989). Released GABA stimulates GABA_*A*_ receptor (GABA_*A*_R) anion channels leading to Cl^–^ inflow driven by iGluR-induced depolarization. Resultant NaCl inflow brings about neuronal swelling (Inoue et al., 2007; Tymianski, 2011) and activation of VSOR (Inoue et al., 2007). Glutamate released from astrocytes activates neuronal metabotropic glutamate receptor (mGluR) and then neuronal VSOR by Ca^2+^ nanodomain-mediated ROS production (Akita and Okada, 2014) (also see Figure 4 in Okada et al., 2019b). VSOR channels thus activated under excitotoxic conditions serve as a swelling-exaggerating, instead of volume-regulatory, Cl^–^ influx pathway, because of depolarization induced by activation of iGluR, thereby causing NVI induction and RVD dysfunction, and then eventually leading to necrotic cell death (Inoue et al., 2007), as summarized in Figure 3C (right panel). Thus, VSOR doubly contributes to excitotoxic neuronal NVI and necrosis by mediating glutamate release from astrocytes and by enhancing Cl^–^ inflow into neurons (Figure 3C).

Regulated or programmed necrosis, which occurs in genetically controlled, but not accidental, manner, includes necroptosis, pyroptosis, and ferroptosis. Necroptosis is dependent on receptor-interacting protein kinase 3 (RIPK3), triggered by activation of death receptors or Toll-like-receptor (TLR) in the presence of caspase-8 inhibition (Green, 2019; Sauler et al., 2019), and regulated by activation of RIPK3 (Kaiser et al., 2013) which induces phosphorylation of mixed lineage kinase domain-like protein (MLKL). Phosphorylated MLKL oligomerizes and translocates to the plasma membrane to form a pore of ∼4 nm diameter (Ros et al., 2017) leading to rapid rupture of the plasma membrane. Necroptosis exhibits marked cell swelling (Chen et al., 2016), but the ionic mechanism of this NVI event remains unexplored. Necroptosis is known to be characterized by intracellular ATP depletion (Henriquez et al., 2008; Vandenabeele et al., 2010) which is possibly induced by ATP consuming process of activation of poly(ADP-ribose)polymerase 1 (PARP1) (Jouan-Lanhouet et al., 2012). Thus, it is likely that ATP depletion is responsible for induction of NVI associated with necroptosis (see Figures 2A,B, right panels).

On the other hand, pyroptosis is inflammatory caspase-dependent programmed necrosis. Pyroptosis is triggered by exposure of cells to bacteria, virus and toxins, called pathogen-associated or danger-associated molecular patterns (PAMPs or DAMPs), which induce formation of multiprotein complexes called inflammasomes in inflammatory cells (Broz and Dixit, 2016). Pyroptosis is regulated by inflammatory caspases (caspase-1, -4, -5, and -11) which mediate cleavage of gasdermin D (GSDMD) into the NH_2_-terminal of GSDMD (GSDMD-N), the oligomer of which forms the non-selective membrane pore (Ding et al., 2016; Sborgi et al., 2016; Ruan et al., 2018). Pyroptosis exhibits discernible, though less markedly compared to necroptosis, cell swelling (NVI) (Fink and Cookson, 2006; Chen et al., 2016). Since pyroptotic inflammasome activation involves K^+^ efflux, TRPM2/V2-mediated Ca^2+^ influx and Cl^–^ channel activation (Tang et al., 2017; Green et al., 2018), it is possible that ionic fluxes are also implicated in the pyroptotic NVI induction. Especially, it is noted that the Cl^–^ channel was shown to be sensitive to VSOR blockers including DCPIB and NPPB (Green et al., 2018) and that flufenamic acid (FFA), which is a known inhibitor of cyclooxygenase (COX), was shown to inhibit inflammasome via blocking VSOR but not via COX-1/COX-2 inhibition (Daniels et al., 2016).

The third type of programmed necrosis is ferroptosis dependent on lipid peroxide. Ferroptosis is triggered by GSH depletion induced by small molecule ferroptosis inducers such as erastin, sorafenib, sulfasalazine bothonine sulphoximine (BSO) and RSL3 (Cao and Dixon, 2016; Sun et al., 2018). This process is regulated by inhibition of GSH-dependent antioxidant enzyme, glutathione peroxidase 4 (GPX4) (Cao and Dixon, 2016; Lei et al., 2019). Ferroptotic cell death is caused by iron-dependent accumulation of lipid peroxides (L-ROS) which give rise to oxidative damage to the cell membrane (Del Re et al., 2019; Lei et al., 2019). No study has been reported as to whether ferroptosis also exhibits cell swelling. It is also warranted to examine whether VSOR is involved in ferroptosis-associated GSH depletion.

## Conclusion and Perspective

In conclusion, VSOR/VRAC activity is essentially involved not only in CVR and protection from cell injury/death but also in induction of apoptotic/necrotic cell death by inducing Cl^–^ outflow or inflow and releasing organic signal molecules. However, their detailed molecular mechanisms remain unexplored.

Urgent issues to be clarified are to provide firm evidence for pore formation by the LRRC8 heteromer, information about the 3-D structure of LRRC8 heterohexamer by cryo-EM studies, and actual roles of VSOR/VRAC in programmed necrosis such as necroptosis, pyroptosis, and ferroptosis. Also, a question to be soon unraveled is what are missing molecular components actually involved in activation and regulation of VSOR/VRAC.

Recently, an essential involvement of VSOR/VRAC in stroke-induced brain damage was clearly demonstrated by *in vivo* studies using conditional LRRC8A-knockout mice (Yang et al., 2019b). In this context, it is noted that accumulating evidence has been provided for neuroprotective effects of VSOR/VRAC blockers on ischemic neuronal damage/death in the brain by *ex vivo* slice experiments (Zhang et al., 2011) and by *in vivo* experiments (Phillis et al., 1997, 1998; Seki et al., 1999; Kimelberg et al., 2000; Feustel et al., 2004; Kimelberg, 2005; Inoue et al., 2007; Zhang et al., 2008; Alibrahim et al., 2013). Thus, more specific, less toxic, and blood-brain barrier-permeable VSOR/VRAC blocking agents are awaited to be developed for clinical use hopefully within the next decade.

## Author Contributions

YO conceived of the project. YO and RS wrote the manuscript (Part 1) with input from KS-N and TN. TN and RS prepared figures. KS-N prepared references. All authors contributed to the article and approved the submitted version.

## Conflict of Interest

The authors declare that the research was conducted in the absence of any commercial or financial relationships that could be construed as a potential conflict of interest.
